# Correlation analysis of serum CCL3, TRACP-5b, and sclerostin with the prognosis of multiple myeloma patients

**DOI:** 10.5937/jomb0-62533

**Published:** 2026-06-16

**Authors:** Cuimei Jiang, Manman Sun, Dashan Gao, Xiaoli Zhang, Jianan Pan

**Affiliations:** 1 Tangshan People's Hospital, Department of Orthopaedics, Tangshan City, China; 2 Huai'an Hospital of Huai'an City (Huai'an Clinical Medical College of Jiangsu University), Department of Hospital Infection, Huai'an District, Huai'an City, China; 3 The First Affiliated Hospital of Zhengzhou University, Department of Oncology, Erqi District, Zhengzhou City, China; 4 The First People's Hospital of Taizhou, Department of Orthopaedics, Zhejiang Province, Affiliated to Wenzhou Medical University, Taizhou City, China

**Keywords:** tartrate-resistant acid phosphatase 5b, Sclerostin, multiple myeloma, prognostic correlation, c-c motif chemokine ligand 3, tartrat-rezistentna kiselinska fosfataza 5b, sklerostin, multipli mijelom, prognostička korelacija, C-C motiv hemokin ligand 3

## Abstract

**Background:**

To investigate the clinical use of Sclerostin, tartrate-resistant acid phosphatase 5b (TRACP-5b), and serum C-C motif chemokine ligand 3 (CCL3) levels in assessing the health and prognosis of patients with multiple myeloma.

**Methods:**

A total of 250 patients with multiple myeloma who visited the hospital between January 2023 and December 2024 were included as the observation group, while 150 healthy individuals evaluated during the same period served as the control group. Serum levels of CCL3, TRACP-5b, and Sclerostin were measured and compared in the observation group before and after treatment. Additionally, serum levels of these markers were analysed in MM patients with different tumour stages and varying degrees of bone destruction. Univariate and multivariate analyses were performed to identify factors associated with death within one year in MM patients. Finally, the predictive efficacy of single and combined detection of serum CCL3, TRACP-5b, and Sclerostin for one-year mortality in multiple myeloma patients was evaluated.

**Results:**

The levels of serum CCL3, TRACP-5b, and Sclerostin in the observation group before and after treatment were significantly greater than those in the control group, and the differences were statistically significant (P&lt;0.05). After treatment, the levels of serum CCL3, TRACP-5b, and Sclerostin in the observation group were significantly lower than those before treatment, and the differences were statistically significant (P&lt; 0.05). There were statistically significant differences in tumour stage, classification of bone destruction, CCL3 level, TRACP-5b level, and Sclerostin level (P&lt;0.05). Multivariate analysis revealed that tumour stage, the degree of bone destruction, the CCL3 level, the TRACP-5b level, and the Sclerostin level were factors influencing death within 1 year in patients with multiple myeloma (P&lt;0.05). The levels of serum CCL3, TRACP-5b, and Sclerostin have relatively high predictive value for death within 1 year in patients with multiple myeloma. The sensitivity of the combined detection of the three indicators was 90.3%, the specificity was 87.4%, and the area under the receiver operating characteristic curve (AUC) was 0.937, which was significantly greater than that of CCL3 (Z = 3 .0 6 1 , P=0.002), TRACP-5b (Z= 3.625, P&lt;0.001), and Sclerostin (Z= 2.579). When tested separately (P= 0.010), there was no statistically significant difference in AUC among the three indicators (P&gt;0.05).

**Conclusions:**

CCL3, TRACP-5b, and Sclerostin are involved in the development and progression of multiple myeloma and have high predictive value for death within 1 year.

## Abstract (sr)

**Uvod:**

Cilj je bio da se ispita klinička primena nivoa sklerostina, tartrat-rezistentne kiseline fosfataze 5b (TRACP-5b) i serumskog C-C motiv hemokina liganda 3 (CCL3) u proceni zdravstvenog stanja i prognoze kod pacijenata sa multiplim mijelomom.

**Metode:**

Za posmatranu grupu izabrano je 250 pacijenata sa multiplim mijelomom koji su posetili bolnicu u periodu od januara 2023. do decembra 2024. godine, dok je kontrolnu grupu činilo 150 zdravih ispitanika koji su u istom periodu evaluirani u bolnici. Upoređivani su nivoi serumske CCL3, TRACP-5b i sklerostina u posmatranoj grupi pre i posle tretmana, kao i među pacijentima sa različitim stadi-jumima tumora i različitim stepenom oštećenja kostiju. Analizirani su jednovarijantni i multivarijantni faktori smrti unutar jedne godine kod pacijenata sa multiplim mijelo-mom, kao i prediktivna efikasnost pojedinačnog i kombino-vanog određivanja serumske CCL3, TRACP-5b i sklerostina za smrt unutar jedne godine.

**Rezultati:**

Nivoi serumske CCL3, TRACP-5b i sklerostina u posmatranoj grupi pre i posle tretmana su bili značajno viši u odnosu na kontrolnu grupu, sa statistički značajnim razlikama (P&lt; 0,05). Nakon tretmana, nivoi serumske CCL3, TRACP-5b i sklerostina u posmatranoj grupi su bili značajno niži nego pre tretmana, što je takođe statistički značajno (P&lt; 0,05). Postojale su statistički značajne razlike u stadijumu tumora, klasifikaciji stepena ostećenja kostiju, nivou CCL3, nivou TRACP-5b i nivou sklerostina (P&lt; 0,05). Multivarijantna analiza je pokazala da su stadijum tumora, stepen oštećenja kostiju, nivo CCL3, nivo TRACP-5b i nivo sklerostina faktori koji utiču na smrt unutar jedne godine kod pacijenata sa multiplim mijelomom (P&lt; 0,05). Nivoi serumske CCL3, TRACP-5b i sklerostina imaju relativno visoku prediktivnu vrednost za smrt unutar jedne godine kod pacijenata sa multiplim mijelomom. Osetljivost kombi-novanog određivanja ova tri indikatora bila je 90,3%, speci-fičnost 87,4%, a povrsina ispod AUC krive 0,937, sto je značajno vise u odnosu na pojedinačne testove CCL3 (Z = 3,061 , P= 0,002), TRACP-5b (Z = 3,625 , P&lt; 0,001) i sklerostina (Z = 2,579 ). Prilikom pojedinačnog testiranja (P= 0,010) nije bilo statistički značajne razlike u AUC između tri indikatora (P&gt; 0,05).

**Zaključak:**

CCL3, TRACP-5b i sklerostin su uključeni u nas-tanak i razvoj multiplog mijeloma i imaju visoku vrednost u predviđanju smrti unutar jedne godine.

## Introduction

A malignant B-cell tumour, multiple myeloma is primarily identified by the proliferation of plasma cells, accounting for 13% of haematological malignancies, and its incidence is increasing [1]. Multiple myeloma-related bone disease is a common complication of multiple myeloma and seriously affects the quality of life of patients. The quality of life for people with multiple myeloma is directly correlated with the extent of bone destruction. Improving the prognosis of patients with multiple myeloma bone disorders requires early identification and therapy [2]. Although imaging examinations provide direct evidence of multiple myeloma-related bone disease, they cannot be used for early, precise quantitative evaluation [3][4][5]. Bone metabolism markers are indicators for evaluating bone metabolism and have been the focus of relatively few studies in multiple myeloma-related bone diseases [6]. Activated osteoclasts are the primary source of tartrate-resistant acid phosphatase 5b (TRACP-5b), which supports bone metabolism and promotes bone resorption [7]. Sclerostin can regulate osteoclast activity via the Wnt signalling pathway, thereby influencing abnormal bone metabolism [8]. Its course is complex, and the prognosis is highly heterogeneous. Bone disease is the most common and destructive complication of this disease [9][10]. In addition to hurting patients' quality of life, osteolysis is strongly linked to the advancement of the illness and a poor prognosis [11].

A thorough understanding of the molecular mechanisms underlying the onset and progression of MM-related bone diseases, and the identification of biomarkers that accurately reflect disease activity and predict patient prognosis, are vital for achieving individualized treatment and improving patient survival. Recent studies [12][13][14] have shown that an imbalance of cytokines and bone metabolism molecules in the bone marrow microenvironment is the key factor driving MM-related bone disease. Among them, C-C motif chemokine ligand 3 (CCL3) is an essential osteoclast-activating factor that can promote osteoclast generation and bone resorption. Tartrate-resistant acid phosphatase 5b (TRACP-5b), a specific marker of osteoclast activation, can directly reflect the degree of bone destruction. Sclerostin is an important negative regulator of bone formation, and its expression may be inhibited in MM. However, existing studies have explored the associations of CCL3, TRACP-5b, or Sclerostin with MM bone disease or disease progression [15]. However, comprehensive assessment of the combined expression levels of these three key factors in serum (representing inflammatory chemotaxis, osteoclast activity, and osteogenic inhibition) and their correlation with the overall prognosis of MM patients (such as overall survival or progression-free survival) remains insufficient [16][17][18].

Therefore, by detecting serum levels of CCL3, TRACP-5b, and Sclerostin in MM patients, their relationships with indicators, such as clinical stage and bone disease severity, can be analysed. The predictive value of individual or combined detection of the three indicators for the prognosis of MM patients can be explored to identify new, more comprehensive serological prognostic indicators that provide a theoretical basis for precise risk stratification and optimized treatment strategies for MM patients.

## Materials and methods

### General information

The observation group consisted of 250 patients with multiple myeloma who presented to our institution between January 2023 and December 2024, 134 men and 116 women. The median age was 52 years, with a range of 38 to 71 years. Seventy-two patients were in stage I, 102 were in stage II, and 76 were in stage III, as determined by the international staging system. The classification was based on the degree of bone destruction: Grade 0 with no bone destruction (70 cases), Grade 1 with widespread osteoporosis or one instance of bone destruction (38 cases), Grade 2 with 2 to 3 instances of bone destruction (86 cases), and Grade 3 with more than 3 instances of bone destruction or pathological fractures or compression fractures (56 cases).

The control group consisted of an additional 150 healthy individuals, 82 men and 68 women. All research subjects provided informed consent and signed the informed consent form. This study was reviewed and approved by the hospital ethics committee [No. 2022-745-48].

### Inclusion and exclusion criteria

Inclusion criteria: Multiple myeloma and multiple myeloma bone disease, meeting the corresponding diagnostic criteria; complete case data; and good compliance.

Exclusion criteria: Patients with other malignant tumours; other haematological diseases; bone and joint diseases; endocrine diseases such as hyperthyroidism, hypothyroidism, and diabetes; a history of traumatic fractures; those who received hormone, calcium, and bisphosphonate treatments within one month before enrolment; and those with intellectual decline or mental disorders.

### Treatment methods

All patients underwent examinations before conventional chemotherapy upon admission and were treated with the bortezomib + thalidomide + dexamethasone (VTD) regimen, the bortezomib + liposomal doxorubicin + dexamethasone (VAD) regimen, or the bortezomib + lenalidomide + dexamethasone (VRD) regimen. None of the patients had received bone marrow transplantation or hematopoietic stem cell therapy. In addition, 4 mg of zoledronic acid should be used for treatment once a month within 2 years after the diagnosis of multiple myeloma and once every 3 months after 2 years. In cases of bisphosphonate-related osteonecrosis of the jaw, the use should be suspended for at least three months.

### Serological index detection

After each research participant was enrolled, 5 mL of fasting elbow venous blood was collected and placed in an anticoagulant tube. The blood was centrifuged for 10 minutes at 3,000 rpm to determine serological index values, and the supernatant was refrigerated at -70°C. TRACP-5b, Sclerostin, and serum CCL3 levels were measured using an enzyme-linked immunosorbent test. The kit was acquired from Shanghai Lianmai Bioengineering Co., Ltd.

### Observation indicators

Serum CCL3, TRACP-5b, and Sclerostin levels were compared between the observation and control groups. Additionally, the levels of these proteins in the observation group were compared before and after therapy. The levels of serum CCL3, TRACP-5b, and Sclerostin in MM patients with different tumour stages and different degrees of bone destruction were compared. Univariate and multivariate analyses of deaths within one year in patients with multiple myeloma were conducted to compare the predictive efficacy of single and combined detection of serum CCL3, TRACP-5b, and Sclerostin for death within one year.

### Statistical processing methods

The statistical program SPSS 20.0 was used for data processing and analysis. x̄±s is the expression for measuring data that follows a normal distribution. Independent sample t tests were used for two-group comparisons. A paired t-test was used to compare the observation group's results before and after therapy. The F test was used to compare multiple groups. Additional pairwise comparisons were conducted using the LSD test. For group comparisons, the χ^2^ test was used, and counts are presented as percentages or as the number of cases. Serum CCL3, TRACP-5b, and Sclerostin levels were examined for their predictive value of death within a year in multiple myeloma patients using receiver operating characteristic (ROC) curves.

## Results

### Comparison of serum CCL3, TRACP-5b, and Sclerostin levels

Serum CCL3, TRACP-5b, and Sclerostin levels were significantly higher in the observation group than in the control group both before and after therapy. Additionally, following treatment, the observation group's serum CCL3, TRACP-5b, and Sclerostin levels were significantly lower than those before treatment (P<0.05), as shown in Table 1.

**Table 1:** Comparison of serum MIP-1α, TRACP-5b, and osteoprotein levels between the observation group and the control group.

Group	n	Time	CCL3 (pg/mL)	TRACP-5b (ng/mL)	Sclerostin (pg/mL)
Observation group	250	Before treatment	133.16±38.04	32.41±9.71	0.68±0.14
		After treatment	76.46±14.21	16.25±3.85	0.35±0.02
Control group	150		47.56±12.45	9.45±2.10	0.24±0.01

### Comparison of serum CCL3, TRACP-5b, and Sclerostin levels in patients with multiple myeloma at different tumour stages

The levels of serum CCL3, TRACP-5b, and Sclerostin in patients with stage III MM were significantly greater than those in patients with stage I and stage II MM. Patients with stage II MM had significantly higher serum levels of CCL3, TRACP-5b, and Sclerostin than patients with stage I MM (P<0.05), as shown in Table 2.

**Table 2:** Comparison of serum MIP-1α, TRACP-5b, and osteopontin levels in patients with multiple myeloma at different tumour stages.

Instalment	n	CCL3 (pg/mL)	TRACP-5b (ng/mL)	Sclerostin (pg/mL)
Phase I	72	88.99±15.69	20.80±5.57	0.55±0.07
Phase II	102	130.41±11.94	32.20±2.83	0.67±0.06
Phase III	76	178.56±19.86	43.79±4.31	0.71±0.01
F		305.227	264.561	245.692
P		<0.001	<0.001	<0.001

### Comparison of serum CCL3, TRACP-5b, and Sclerostin levels in MM patients with different degrees of bone destruction

With increasing bone destruction grade in patients with multiple myeloma, the levels of serum CCL3, TRACP-5b, and Sclerostin significantly increased (P<0.05), as shown in Table 3.

**Table 3:** Comparison of serum MIP-1α, TRACP-5b, and osteopontin levels in patients with multiple myeloma of different grades of bone destruction.

Grading	n	CCL3 (pg/mL)	TRACP-5b (ng/mL)	Sclerostin (pg/mL)
Level 0	70	88.35±15.43	20.61±5.42	0.55±0.07
Level 1	38	117.54±4.21	29.10±0.91	0.63±0.05
Level 2	86	142.19±11.87	35.06±2.74	0.60±0.06
Level 3	56	185.80±17.85	45.59±3.69	0.84±0.00
F		275.774	238.909	212.928
P		<0.001	<0.001	<0.001

### Univariate analysis of mortality within one year in patients with multiple myeloma

All patients were followed for one year. Among them, 80 died, and 170 survived. Tumour stage, degree of bone degradation categorization, CCL3 level, TRACP-5b level, and Sclerostin level were all statistically different between the death and survival groups (P<0.05). However, the survival and nonsurvival groups did not differ significantly in sex, body mass index (BMI), or chemotherapy regimens (P>0.05), as shown in Table 4.

**Table 4:** Univariate analysis of mortality within 1 year in patients with multiple myeloma.

Group	n	Male	BMI <br>(kg/m^2^)	Tumour <br>staging			Grading of bone <br>destruction degree				Chemotherapy <br>regimen		
				Phase I	Phase II	Phase III	Level 0	Level 1	Level 2	Level 3	VTD	VAD	VRD
Survival group	170	82 (48.24)	23.11±2.12	54	78	38	66	30	60	14	28	70	72
Death group	80	52 (65.00)	22.75±1.66	18	24	38	4	8	26	42	22	30	28
χ2/t		2.430	1.185	7.892			37.638				3.008		
P		0.112	0.232	0.010			<0.001				0.226		
Group		n		CCL3 (pg/mL)			TRACP-5b (ng/mL)				Sclerostin (pg/mL)		
Survival group		170		119.32±31.47			29.71±8.10				0.63±0.01		
Death group		80		162.36±34.37			38.24±10.55				0.77±0.14		
χ2/t				6.918			4.896				6.940		
P				<0.001			<0.001				<0.001		

### Analysis of factors influencing death within one year in patients with multiple myeloma

Multivariate analysis of indicators with statistically significant differences identified via univariate analysis revealed that tumour stage, classification of degree of bone destruction, CCL3 level, TRACP-5b level, and Sclerostin level were independent predictors of death within one year in patients with multiple myeloma (P<0.05), as shown in Table 5.

**Table 5:** Multivariate analysis of mortality within 1 year in patients with multiple myeloma.

Influencing factors	β	SE	Wald χ2	P	Exp (β)	95% CI
Tumour staging	2.891	1.239	5.504	0.012	0.058	0.008~0.624
Grading of bone <br>destruction degree	3.497	0.973	12.971	<0.001	32.914	4.911~220.222
CCL3	0.045	0.016	10.800	0.004	1.045	1.010~1.062
TRACP-5b	0.090	0.044	5.617	0.011	1.105	1.010~1.197
Sclerostin	20.506	6.632	9.530	0.005	0.008	1.791~3.592

### Predictive efficacy of serum CCL3, TRACP-5b, and Sclerostin levels for death within one year in patients with multiple myeloma

The levels of serum CCL3, TRACP-5b, and Sclerostin have relatively high predictive value for death within 1 year in patients with multiple myeloma. To determine whether death occurred within a year, a multivariate logistic regression analysis was performed. The equation Y=0.04x xmp-1α + 0.11x XTRACP-5b + 13.88xX Sclerostin -19.31 was obtained to establish a thorough detection indication. The sensitivity of the combined detection of the three indicators was 90.3%, and the specificity was 87.4%. The area under the curve (AUC) was 0.937, substantially higher than the AUCs of the independently discovered Sclerostin (Z=2.579, P=0.010), TRACP-5b (Z=3.625, P<0.001), and CCL3 (Z=3.061, P=0.002). The three indicators' AUCs did not differ statistically significantly (P>0.05), as shown in Table 6.

**Table 6:** Predictive efficacy of serum MIP-1α, TRACP-5b, and osteopontin levels for mortality within one year in patients with multiple myeloma.

Indicator	Truncation value	Sensitivity (%)	Specificity (%)	AUC	95% CI
CCL3	163.35 pg/mL	60.3	92.2	0.817	0.738~0.871
TRACP-5b	38.71 ng/mL	62.8	91.1	0.768	0.684~0.839
Sclerostin	0.75 pg/mL	67.8	94.4	0.845	0.769~0.904
3 Joint Projects	-	90.3	87.4	0.937	0.878~0.974

## Discussion

Multiple myeloma-related bone disease is a complication of multiple myeloma. Multiple myeloma and the bone marrow matrix are closely connected [19]. The composition of the bone marrow matrix is influenced by multiple myeloma cells, which promote multiple myeloma growth and cause extensive osteolytic lesions [20]. Normal bone metabolism is closely related to the balance between osteoblasts and osteoclasts. The results of this study revealed that tumour stage, bone destruction degree, CCL3 level, TRACP-5b level, and Sclerostin level are factors influencing death within one year in patients with multiple myeloma. Studies [21][22][23] have shown that Numerous cytokines, including tumour necrosis factor, interleukin-3, and CCL3, are secreted by multiple myeloma cells and have an inhibitory influence on osteoblast activity, mostly by preventing stromal cells from transforming into osteoblasts [24]. The serum CCL3 level increases with tumour stage and the degree of bone destruction in multiple myeloma, indicating that it is an indicator of disease severity [25]. The increased degree of bone destruction promotes the proliferation of multiple myeloma and bone destruction, forming a vicious cycle. Moreover, after CCL3 binds its receptor, it activates osteoclasts, leading to enhanced metabolism and osteolytic destruction of bone [26]. Their serum CCL3 level can predict multiple myeloma patients' prognosis, since the death group's level was much higher than the survival group's [27]. When the cut-off value for serum CCL3 was 163.35 pg/mL, its sensitivity was 60.3%, its specificity was 92.2%, and its AUC was 0.817, indicating that serum CCL3 levels are highly predictive of death within 1 year in patients with multiple myeloma.

Bone metabolism plays a significant role in both osteoclasts and osteoblasts [28][29][30]. Disruption of the balance between the two can lead to bone diseases. TRACP-5b is a cytokine secreted by osteoclasts. When osteoclasts are activated, TRACP-5b is secreted outside the cells. Therefore, serum TRACP-5b levels are an important indicator of osteoclast activity and the severity of osteolysis. The observation group's serum TRACP-5b level was significantly higher before and after treatment than the control group's, and it was significantly lower after treatment than before, suggesting a relationship between TRACP-5b levels and the onset and progression of multiple myeloma. The serum TRACP-5b level increases with tumour stage and the degree of bone destruction, indicating that it is an important indicator of multiple myeloma severity. Patients with multiple myeloma have elevated serum TRACP-5b levels, which are positively connected with the disease's severity and early indicators of osteolytic damage and tumour spread [31]. When bone metastasis occurs in tumours, the activity of osteoclasts increases significantly, and the level of serum TRACP-5b also increases significantly. It has been used to monitor bone metastasis in prostate cancer patients, has high sensitivity and specificity, and is considered a potential replacement for bone scan examinations [32]. The sensitivity for predicting death within a year in multiple myeloma patients was 62.8%, the specificity was 91.1%, and the AUC was 0.768 at the cut-off value of 38.71 ng/mL. Serum TRACP-5b levels in the death group were significantly higher than those in the survival group. These results suggest that serum TRACP-5b levels are a significant predictor of prognosis in multiple myeloma patients.

Sclerostin is an essential cytokine secreted by osteoblasts and plays a significant role in bone metabolism. In the early stage of multiple myeloma, it affects osteoblast differentiation via the classical Wnt signalling pathway and positively regulates this process. As the tumour burden increases and reaches a certain level, it promotes the secretion of Sclerostin by myeloma cells, which inhibits Wnt signalling and thereby causes multiple myeloma-related bone disease [33]. Although the observation group's blood Sclerostin level was higher than the control group's both before and after treatment, it was notably lower after treatment than before, indicating that Sclerostin is associated with the occurrence and development of multiple myeloma. The results of this study revealed that the level of serum osteoporosis increases with the tumour stage of multiple myeloma and the degree of bone destruction [34]. The serum osteoclastic protein level in the nonsurviving group was significantly greater than that in the surviving group. Moreover, when the cut-off value of serum osteoclastic protein was 0.75 pg/mL, the sensitivity for predicting death within one year in multiple myeloma patients was 67.8%, the specificity was 94.4%, and the AUC was 0.845. The results of this study show that the combined detection of serum CCL3, TRACP-5b, and Sclerostin levels has greater predictive efficacy for death within one year in patients with multiple myeloma. Its sensitivity is 90.3%, its specificity is 87.4%, and its AUC is 0.937, all of which are higher than those of the single indicators.

## Conclusion

The development and occurrence of multiple myeloma are influenced by CCL3, TRACP-5b, and Sclerostin, which also have a significant predictive value for death within a year in patients with the disease.

## Dodatak

### Authors' contribution

Cuimei Jiang and ManMan Sun are the first coauthors of this study.

### Conflict of interest statement

All the authors declare that they have no conflict of interest in this work.

## References

[1] Maura F, Rajanna A R, Ziccheddu B, Poos A M, Derkach A, Maclachlan K, Durante M, Diamond B, Papadimitriou M, Davies F, Boyle E M, Walker B, Hultcrantz M, Silva A, Hampton O, Teer J K, Siegel E M, Bolli N, Jackson G H, Kaiser M, Pawlyn C, Cook G, Kazandjian D, Stein C, Chesi M, Bergsagel L, Mai E K, Goldschmidt H, Weisel K C, Fenk R, Raab M S, Van Rhee F, Usmani S, Shain K H, Weinhold N, Morgan G, Landgren O (2024). Genomic Classification and Individualized Prognosis in Multiple Myeloma. J Clin Oncol.

[2] Tavakoli Pirzaman A, Ebrahimi P, Hasanpour A H, Shakeri M, Babajani B, Pourali Ganji Z, Babaei H, Rahmati A, Hosseinzadeh R, Doostmohamadian S, Kazemi S (2023). miRNAs and Multiple Myeloma: Focus on the Pathogenesis, Prognosis, and Drug Resistance. Technol Cancer Res Treat.

[3] Sun C, Zhang W, Liu H, Ding Y, Guo J, Xiong S, Zhai Z, Hu W (2024). Identification of a novel lactylation-related gene signature predicts the prognosis of multiple myeloma and experiment verification. Sci Rep.

[4] Thakral B, Vijayanarayanan A, Medeiros L J, Lin P (2025). Cytogenetic and molecular aberrations at diagnosis and in prognosis of multiple myeloma. Semin Diagn Pathol.

[5] Seegmiller A C (2025). Flow cytometry in the diagnosis and prognosis of multiple myeloma. Semin Diagn Pathol.

[6] Martello M, Solli V, Mazzocchetti G, Solimando A G, Bezzi D, Taurisano B, Kanapari A, Poletti A, Borsi E, Armuzzi S, Vigliotta I, Pistis I, Desantis V, Marzocchi G, Rizzello I, Pantani L, Mancuso K, Tacchetti P, Testoni N, Nanni C, Zamagni E, Cavo M, Terragna C (2024). High level of circulating cell-free tumor DNA at diagnosis correlates with disease spreading and defines multiple myeloma patients with poor prognosis. Blood Cancer J.

[7] Martinez-Cordero H, Pena C, Schutz N P, Bove V, Villano F, Beltran C, Donoso J, Lopez-Vidal H, Roa Salinas M A, Soto P, Ochoa P, Duarte P, Remaggi G, Corzo A, Shanley C, Lopresti S, Orlando S, Verri V, Quiroga L D, Fantl D, Ramirez J, Ospina-Idarraga A, Idrobo H, Quintero G, Gomez R, Cantu-Martinez O, Gomez-Almaguer D, Ruiz-Arguelles G J, Galvez-Cardenas K M, Salazar L A, Novoa-Caicedo I, Fuentes-Lacouture M C, Spirko P, Arbelaez M I, Pereira M, Valdes J, Vasquez J, von Glasenapp A, Riva E, GELAMM (Grupo de Estudio Latinoamericano de Mieloma Multiple) (2023). Patients Age 40 Years and Younger With Multiple Myeloma Have the Same Prognosis as Older Patients: An Analysis of Real-World Patients' Evidence From Latin America. JCO Glob Oncol.

[8] Liang D, Li X, Bai S, Wang Q, Zeng M, Feng D, Lu B, Li X, Sun Z, Li J, Zhou H, Zhang J, Chen X, Xia Z, Liang Y, Wang H (2024). Clinical Outcome of Induction Treatment in the Era of Novel Agents and the Impact of the Number of High-Risk Cytogenetic Abnormalities (HRA) on Prognosis of Patients With Newly Diagnosed Multiple Myeloma (NDMM): Insights From a Multicenter Study. Cancer Med.

[9] Shen N, Zhang J, Xia Y, Shen X X, Wang J, Jin Y Y, Zhang R, Li J Y, Chen L J (2023). Clinical characteristics and prognosis of newly diagnosed multiple myeloma patients with FGFR3 gene mutations. Zhonghua Xue Y Xue Za Zhi.

[10] Yu K, Li J, Chen D, Huan W (2023). The Correlation of miR-671-5p with Diagnosis and Prognosis in Multiple Myeloma. Clin Lab.

[11] Solmaz S, Uzun O, Sevindik O G, Demirkan F, Ozcan M A, Ozsan G H, Alacacıoglu I (2023). The effect of haemoglobin, albumin, lymphocyte and platelet score on the prognosis in patients with multiple myeloma. Int J Lab Hematol.

[12] Wu L, Zheng Y, Liu J, Luo R, Wu D, Xu P, Wu D, Li X (2021). Comprehensive evaluation of the efficacy and safety of LPV/r drugs in the treatment of SARS and MERS to provide potential treatment options for COVID-19. Aging (Albany NY).

[13] Chen H, Zhao Y, Zhang Z, Xie Y, Jin M (2025). Immuno-phenotypic characteristics and prognostic value of peripheral blood circulating plasma cells in patients with newly diagnosed multiple myeloma. J Med Biochem.

[14] Tang S, Long X, Li F, Jiang S, Fu Y, Liu J (2025). Identification of RUVBL2 as a novel biomarker to predict the prognosis and drug sensitivity in multiple myeloma based on ferroptosis genes. Hematology.

[15] Li J R, Parthasarathy A K, Kannappan A S, Arsang-Jang S, Dong J, Cheng C (2024). Characterization of driver mutations identifies gene signatures predictive of prognosis and treatment sensitivity in multiple myeloma. Oncologist.

[16] Wu L, Zhong Y, Wu D, Xu P, Ruan X, Yan J, Liu J, Li X (2022). Immunomodulatory Factor TIM3 of Cytolytic Active Genes Affected the Survival and Prognosis of Lung Adenocarcinoma Patients by Multi-Omics Analysis. Biomedicines.

[17] Bao L, Wang Y T, Lu M Q, Chu B, Shi L, Gao S, Fang L J, Xiang Q Q, Ding Y H, Liu X, Zhao X, Wang M Z, Chen Y, Hu W K (2023). Vitamin D deficiency linked to abnormal bone and lipid metabolism predicts high-risk multiple myeloma with poorer prognosis. Front Endocrinol (Lausanne).

[18] Zhong H, Huang D, Wu J, Chen X, Chen Y, Huang C (2023). 18F FDG PET/CT based radiomics features improve prediction of prognosis: multiple machine learning algorithms and multimodality applications for multiple myeloma. BMC Med Imaging.

[19] Wu L, Liu Q, Ruan X, Luan X, Zhong Y, Liu J, Yan J, Li X (2023). Multiple Omics Analysis of the Role of RBM10 Gene Instability in Immune Regulation and Drug Sensitivity in Patients with Lung Adenocarcinoma (LUAD). Biomedicines.

[20] Zhang Y, Pan J, Kang H, Peng S, Tung T H, Shen B (2024). Prognosis of concurrent renal impairment at diagnosis of multiple myeloma: a systematic review. Ann Med.

[21] Jiang J Y, Yao F Y, Liu J, Wang X L, Huang B, Zhong F M, Wang X Z (2024). A Novel Necroptosis-Related Signature Can Predict Prognosis and Chemotherapy Sensitivity in Multiple Myeloma. Technol Cancer Res Treat.

[22] Wang J H, Xiang P, Zhao E J, Liu L N, Liu Y Z, Liang L J, Cui Y S, Fang B J (2025). Impact of type 2 and steroid-Induced diabetes mellitus on prognosis in patients with multiple myeloma. World J Surg Oncol.

[23] Zhang Y Q, Zhao J S, Wei X F, Feng Y F, Fu Y, Chen Q L, Zhang Q K (2025). Clinical Characteristics and Prognosis of Patients with IgD Multiple Myeloma. Zhongguo Shi Yan Xue Ya Xue Za Zhi.

[24] Wu L, Zheng Y, Ruan X, Wu D, Xu P, Liu J, Wu D, Li X (2022). Long-chain noncoding ribonucleic acids affect the survival and prognosis of patients with esophageal adenocarcinoma through the autophagy pathway: construction of a prognostic model. Anticancer Drugs.

[25] Uzman H, Kaçmaz M (2024). The role of pentraxin 3 and oxidative status in the prognosis of multiple myeloma. J Investig Med.

[26] Qiao X, Song Z, Geng L, Xing L, Wang Y (2025). Investigating the role of tumor cell heterogeneity and angiogenesis genes in the prognosis of multiple myeloma. Front Immunol.

[27] Ji J, Guo R, Ma J, Cui Y, Li Y, Sun Z, Li J, Fan L, Qu X (2023). Liquid extramedullary disease in multiple myeloma strongly predicts a poor prognosis and is associated with bortezomib resistance gene upregulation. Clin Chim Acta.

[28] Yan H, Ding Y, Dai W, Wang H, Qin H, Zhai Z, Tao Q (2025). Identification and construction of a novel NET-related gene signature for predicting prognosis in multiple myeloma. Clin Exp Med.

[29] Wu L, Zhong Y, Yu X, Wu D, Xu P, Lv L, Ruan X, Liu Q, Feng Y, Liu J, Li X (2022). Selective poly adenylation predicts the efficacy of immunotherapy in patients with lung adenocarcinoma by multiple omics research. Anticancer Drugs.

[30] Lei A, Liao X, Zhu P, Xiong M (2023). The significance of autologous hematopoietic stem cell transplantation on immunoglobulin reconstitution and prognosis in elderly patients with multiple myeloma. Hematology.

[31] Xu Y, Cao X, Zhou H, Xu H, Chen B, Bai H (2025). Identifying potential prognosis markers in relapsed multiple myeloma via integrated bioinformatics analysis and biological experiments. Curr Res Transl Med.

[32] Wu L, Li H, Liu Y, Fan Z, Xu J, Li N, Qian X, Lin Z, Li X, Yan J (2024). Research progress of 3D-bioprinted functional pancreas and in vitro tumor models. International Journal of Bioprinting.

[33] You H, Yao W, Yan L, Zhai Y, Yan Z, Shang J, Yan S, Shi X, Jin S, Ge X, Shen H, Pan J, Wu D, Fu C (2025). Poor prognosis of newly diagnosed multiple myeloma patients with 1p32.3 deletion in single monoallelic deletion and/or in main clone. Br J Haematol.

[34] Wang F, Shen C (2025). Impact of liquid liquid phase separation- and immune-related gene signatures on multiple myeloma prognosis: focus on DDX21 and EZH2. Hematology.

